# The Clinics at the Dental Department of the University of Maryland

**Published:** 1883-03

**Authors:** 


### EDITORIAL, ETC.

---

*The Clinics of Dr. W W. Evans at the Dental Department of the University of Maryland:*—During the Session which closed on the 15th of the present month (March) some very interesting Dental Clinics have been given at the University by a number ot experienced operators which we will notice from time to time in future issues of the JOURNAL. Among the first in the order of date were clinics by Dr. Evans "on Filling Teeth and the Manipulation of Celluloid."

Dr· Evans presented to the class a "Plugger" of his own invention, which was not only entirely new and novel in construction, but the working of which packed the gold so rapidly and smoothly when attached to the Dental Engine, as to command the admiration and approval of the class and the visitors n attendance. This "Plugger" can be attached to any dental motor, the blow being thoroughly controlled by the finger, stopped at will to pick up gold, and producing such a delicate elastic blow as to commend it above all other "Pluggers" in use. A very marked characteristic of this instrument is its agreeable sensation to the patient, and its effective working of the gold.

At subsequent Clinics Dr. Evans demonstrated the use of his new Vulcanizer and Heater for Rubber and Celluloid work, which for safety and simplicity cannot be excelled. The following description of the apparatus will convey a good idea of its merits :

In the first place, we have two separable cups united concentrically to form a water aud steam space, the inner cup forming the dry oven, or vulcanizing chamber, and being sufficiently deep for two flasks ; the strength of the cups is carefully tested before they are united. The top is separate, and in the manipulation of celluloid simply rests loosely on the boiler; but in vulcanizing rubber, when steam enters the inner chamber, it is fastened down by several lugs attached to the top, dropping into slots in a ring connected with the boiler and working on inclined planes ; with these it is securely backed by a slight lateral movement. Three balls running through this top and screwing into a triangular platten furnish the means by which the flask, or flasks, is, or are closed. As the heads of the bolts are spherical, the instant you bind them tight in these cup-like sockets—and for this to happen the flask must be closed and will be flush against the top—you render the joints steam-tight, thus doing away with packing-boxes and caps. Another very important feature is that whatever pressure you may choose to exert upon the bolts in closing the flask comes upon the *top alone,* and by this means is avoided that severe strain to which some boilers are subjected by the *down-plungers.* No lock-flasks are needed ; you can view your work at any moment; cool off when you like, and all without changing the temperature of your boiler. A thermometer, steam-valve connecting boiler and chamber, and a simple safety-valve, adjustable at any desired heat or pressure, complete the apparatus, with the exception of a jacket having a transparent door in the front, through which the flame of the lamp may be watched.

